# A Robust Inner and Outer Loop Control Method for Trajectory Tracking of a Quadrotor

**DOI:** 10.3390/s17092147

**Published:** 2017-09-19

**Authors:** Dunzhu Xia, Limei Cheng, Yanhong Yao

**Affiliations:** Key Laboratory of Micro-Inertial Instrument and Advanced Navigation Technology, Ministry of Education, School of Instrument Science and Engineering, Southeast University, Nanjing 210096, China; 220152675@seu.edu.cn (L.C.); 220142708@seu.edu.cn (Y.Y.)

**Keywords:** quadrotor unmanned aerial vehicle (UAV), inner and outer loop, SMC, trajectory tracking, UWB

## Abstract

In order to achieve the complicated trajectory tracking of quadrotor, a geometric inner and outer loop control scheme is presented. The outer loop generates the desired rotation matrix for the inner loop. To improve the response speed and robustness, a geometric SMC controller is designed for the inner loop. The outer loop is also designed via sliding mode control (SMC). By Lyapunov theory and cascade theory, the closed-loop system stability is guaranteed. Next, the tracking performance is validated by tracking three representative trajectories. Then, the robustness of the proposed control method is illustrated by trajectory tracking in presence of model uncertainty and disturbances. Subsequently, experiments are carried out to verify the method. In the experiment, ultra wideband (UWB) is used for indoor positioning. Extended Kalman Filter (EKF) is used for fusing inertial measurement unit (IMU) and UWB measurements. The experimental results show the feasibility of the designed controller in practice. The comparative experiments with PD and PD loop demonstrate the robustness of the proposed control method.

## 1. Introduction

A quadrotor is a kind of unmanned aerial vehicle which consists of four fixed-pitch propellers attached to motors mounted in a square crossing rack. Due to its special structure, a quadrotor is capable of vertical takeoff and landing, as well as hovering. High maneuverability and flexibility make it possible for special applications such as search missions, monitoring and anti-poaching missions [1,2]. 

In order to realize autonomous quadrotor flight, a variety of control methods have been developed to realize trajectory tracking. Bouabdallah modeled the quadrotor with Euler-lagrange formalism and Euler-Newton formalism [1]. Some linear techniques (PID, linear quadratic regulator (LQR)) and nonlinear techniques (backstepping, sliding mode control and fuzzy logic control) have been applied to the control of quadrotor on this basis [3,4]. Besides, He transformed the nonlinear model to a linear model and proposed an internal model control method to realize the trajectory tracking [5]. Nicol et al. proposed a neural network controller for robust trajectory tracking of a quadrotor [6]. González-Vázquez et al. introduced the theory of singularly perturbed systems to the design of trajectory tracking [7]. However, all these approaches are based on Euler angles. The attitude model is obtained on the assumption that roll and pitch are close to zero. Tracking complicated trajectories becomes a challenge for the quadrotor. Tayebi et al. proposed a quaternion-based feedback control strategy for attitude stabilization of a quadrotor as an improvement [8,9,10]. The quaternion is double-covering to the rotation matrix. In order to avoid the singularity and redundancy from Euler angles and quaternion, Lee et al. developed a geometric tracking controller on the special Euclidean group. Results showed the hybrid controller can implement complicated acrobatic maneuvers [11,12,13]. The geometric control method provides a unique perspective to solve the control problem and can avoid the singularity and complexity when using local coordinate [14]. The method is feasible for tracking complicated trajectory. 

Xu et al. proposed a sliding mode control approach to stabilize the under-actuated subsystem (UAS) [15]. Xiong et al. separated the quadrotor system into UAS and fully actuated subsystem (FAS) and designed a control algorithm for FAS and UAS [16]. Meanwhile, most researches separated the system into two cascaded loops. The outer loop governed translational motion and the inner loop stabilized the attitude. The inner-outer loop structure has a more explicit meaning and is easier to design and to tune [17,18]. 

The modeling inaccuracy and external disturbances would give rise to another challenge for accurate tracking trajectory of the quadrotor [19]. SMC has been developed to compensate for parameter uncertainty and bounded disturbances in many applications [20]. However, the chattering effect will be introduced into a system while implementing the SMC. The simplest way to reduce chattering is to replace the sign function with continuous functions such as saturation and hyperbolic tangent function [21]. In addition, high order sliding mode (HOSM) techniques have been proposed to reduce chattering and improve the control performance. The HOSM techniques for a relative degree *r* system cannot reduce the chattering substantially [22]. Then continuous terminal sliding mode control is presented to use on a relative degree *r* system while the chattering is entirely eliminated [23,24]. Considering that chattering effects can be reduced or even eliminated, SMC outperforms traditional control methods in terms of convergence rate and robustness in the presence of model uncertainty and disturbances. 

In our work, we utilize an inner-outer loop structure. The outer loop governs the translational motion and generated reference rotation matrix for the inner loop together with the yaw angle. For the inner loop, the SMC law based on rotation matrix is designed, which frees the quadrotor from the constraints of small-angle flight. The outer loop is designed through SMC. The usage of SMC speeds up the response speed and improves the robustness of the system. The effects of parameters on the system have been studied. The effectiveness of the proposed controller is verified by tracking three representative trajectories. Comparative simulations in two cases are conducted to validate the robustness of the proposed controller. The proposed controller is also validated experimentally. 

The paper is organized as follows: the next section describes the dynamic model of the quadrotor with disturbances. In Section 3, we develop a position controller as well as an attitude controller to achieve trajectory tracking. In Section 4, simulations are conducted to verify the effectiveness of the designed controllers. In Section 5, an experiment is carried out to verify the proposed method. In Section 6, conclusions are presented.

## 2. Dynamic Model

**Figure 1:**
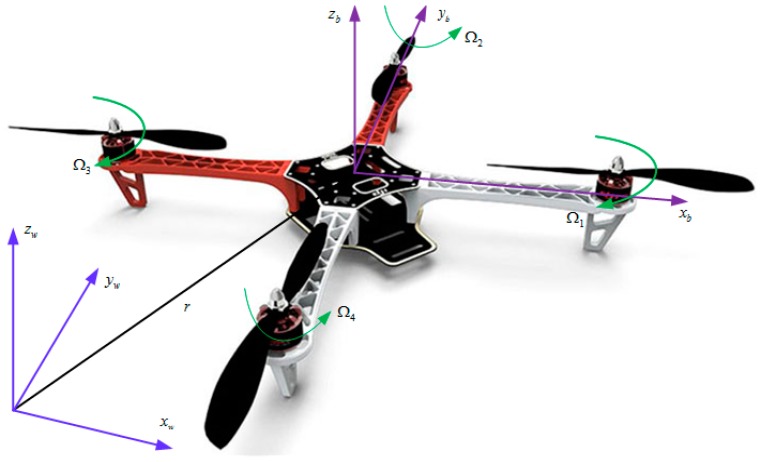
The structure of quadrotor and its coordinate system.

A quadrotor can be regarded as a rigid body. Rigid motion consists of rotation motion and translation motion. Consider the world frame W, as well as the body frame illustrated in Figure 1, the posture of the quadrotor can be described by the relative orientation between the world frame and the body frame. The rotation matrix R∈SO(3) from the body frame to the world frame is utilized to show the posture of quadrotor. Meanwhile, position and velocity in the world frame is denotes by $p\in {\mathbb{R}}^{3}$, $v\in {\mathbb{R}}^{3}$, respectively. The translation and rotation of the quadrotor satisfy:

(1) $$\begin{array}{l} \dot{p}=v\\ \dot{R}=R\hat{\omega } \end{array}$$

where ω denotes the angular velocity of quadrotor in the body frame, $\hat{\cdot }$ represents the hat map from ${\mathbb{R}}^{3}$ to its skew-symmetric matrix. 

According to Newton-Euler formalism, the translational motion of the center of quadrotor in the world frame and the rotational motion of quadrotor in the body frame are as follows:

(2) $$\left(\begin{array}{cc} mE_{3\times 3} & 0\\ 0 & I \end{array}\right)\left(\begin{array}{l} \dot{v}\\ \dot{\omega } \end{array}\right)+\left(\begin{array}{c} 0\\ \omega \times I\omega \end{array}\right)=\left(\begin{array}{l} F\\ M \end{array}\right)$$

where *m* is the mass of the quadrotor. ***I*** is the moment of inertia. ***F*** denotes net force vector in the world frame and ***M*** denotes the moment vector in the body frame. 

Based on the mechanical analysis, the quadrotor is subject to gravity and thrust. When the quadrotor is in actual flight, it is also influenced by drag. Due to the special structure of the quadrotor, the thrust is along z-axis of the body, the amplitude of thrust is denoted by *T*. Therefore, the net force acting on the quadrotor in world frame is given by:

(3) $$F=RTz_{w}-mgz_{w}-K\dot{p}$$

where $T=\sum_{i=1}^{4}T_{i}$, $T_{i}$ is the *i*-th thrust. K represents the drag coefficient matrix. $z_{w}={\left(0,0,1\right)}^{T}\in {\mathbb{R}}^{3}$.

When ignoring body and propeller gyro effects, the moment vector *M* can be written as:

(4) $$M=\left(\begin{array}{c} (T_{2}-T_{4})l\\ (T_{3}-T_{1})l\\ Q_{1}-Q_{2}+Q_{3}-Q_{4} \end{array}\right)$$

where $Q_{i}$ represents the *i*-th counter torque, l represents the distance from the axis of rotation of motors to the center of quadrotor. 

In order to express easily in the simulation, the intermediate variables ${\left(u_{1},u_{2},u_{3},u_{4}\right)}^{T}$ are introduced to represent ${\left(T,M^{T}\right)}^{T}$ as:

(5) $$\left(\begin{array}{l} u_{1}\\ u_{2}\\ u_{3}\\ u_{4} \end{array}\right)=\left(\begin{array}{c} T_{1}+T_{2}+T_{3}+T_{4}\\ (T_{2}-T_{4})l\\ (T_{3}-T_{1})l\\ Q_{1}-Q_{2}+Q_{3}-Q_{4} \end{array}\right)$$

## 3. Trajectory Tracking Control

**Figure 2:**
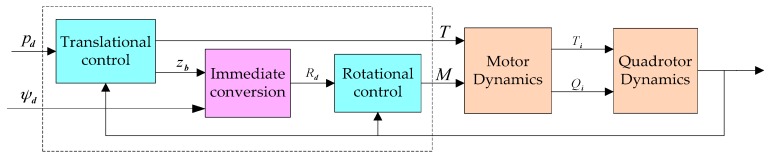
Control structure diagram.

From the analysis above, we can derive that the system is a highly-coupled, under-actuated and nonlinear system and can be divided into two subsystems: an attitude system and a position system. Meanwhile, we can note that the rotational motion is independent of the position, but the translational motion is dependent on the rotational motion. From Equation (3), the *z* axis of the body can be determined once the position of the quadrotor is known. Besides, provided that the yaw angle is given, the attitude of the body can be uniquely determined. In other words, the motion of the quadrotor can be obtained once the position and yaw angle are given. The overall control structure diagram is shown in Figure 2. 

### 3.1. Trajectory Tracking Control

First, we define $p_{d}={\left(x,y,z\right)}^{T}$ and $\psi_{d}$ as the desired trajectory. Now we define the position error and velocity error as:

(6) $$\begin{array}{l} e_{p}=p_{d}-p\\ e_{v}={\dot{p}}_{d}-\dot{p} \end{array}$$

Next, let’s consider the switching function:

(7) $$s=ce_{p}+{\dot{e}}_{p}$$

Then we design the control law:

(8) $$\dot{s}=-ks-\eta sgn(s)$$

where k and η are positive diagonal matrices.

Differentiating Equation (7) and substituting Equations (3) and (6), then:

(9) $$\dot{s}=c{\dot{e}}_{p}+{\ddot{p}}_{d}-(RTz_{w}/m-K\dot{p}/m-gz_{w})$$

Equating Equation (8) with Equation (9), the desired thrust *T* can be derived as follows:

(10) $$T=(m(c{\dot{e}}_{p}+ks+\eta sgn(s)+{\ddot{p}}_{d})+mgz_{w}+K\dot{p})\cdot Rz_{w}$$

where x⋅y is inner product for all $x,y\in {\mathbb{R}}^{3}$. 

Meanwhile, the direction of thrust should be:

(11) $$z_{b}=\frac{c{\dot{e}}_{p}+ks+\eta sgn(s)+{\ddot{p}}_{d}+gz_{w}+K\dot{p}/m}{\left\|c{\dot{e}}_{p}+ks+\eta sgn(s)+{\ddot{p}}_{d}+gz_{w}+K\dot{p}/m\right\|}$$

Provided that the desired position is given, the thrust is designed as Equations (10) and (11). Then the system stabilizes the zero equilibrium of position error exponentially after a finite time.

**Proof:**  In order to verify the designed control law can guarantee the stability of the system, we select the Lyapunov function candidate $V=\frac{1}{2}s^{T}s$, differentiating V and substituting Equation (8):

(12) $$\begin{array}{ll} \dot{V}=s^{T}\dot{s} & =s^{T}(-ks-\eta sgn(s))\\ & =-s^{T}ks-s^{T}\eta sgn(s)\\ & \leq -ks^{T}s \end{array}$$

$\dot{V}\leq 0$ can be proved while k and η are positive diagonal matrices. Therefore, the system is stable and the sliding surface converges to zero exponentially. The stability of outer loop is proved. ☐

Since $s=ce_{p}+{\dot{e}}_{p}$, $e_{p}$ will exponentially converge to zero after a finite time. In other words, the actual position will converge to the desired position. Here the convergence rate is determined by c,k,η. In order to avoid chattering, the sign function is replaced by a saturation function.

Suppose the yaw angle is given, $y_{b}$ and $x_{b}$ can be obtained when the inertial frame rotates to the body frame following the sequence of Z−X−Y. Given the yaw angle $\psi_{d}$, the rotation matrix expressed in the form of coordinate axis can be written as:

(13) $$R_{d}=\left[\frac{x_{f}\times z_{b}}{\left\|x_{f}\times z_{b}\right\|}\times z_{b},\frac{x_{f}\times z_{b}}{\left\|x_{f}\times z_{b}\right\|},z_{b}\right]$$

where $x_{f}={\left(\cos\psi_{d},\sin\psi_{d},0\right)}^{T}$, the singularity where $x_{f}$ is parallel to $z_{b}$ may exist only when the pitch angle or roll angle reaches to 90°. The probability of such a situation is small. In our work, we suppose that we will not encounter the singularity.

### 3.2. Attitude Control

Attitude control is the core of the overall control system. It keeps the orientation of the quadrotor to the desired values precisely. We define the attitude error as follows:

(14) $$R_{e}=R_{d}^{T}R$$

The attitude error represents the relative rotation from the body frame to the reference body frame. Based on the attitude error, we further define angular velocity error as:

(15) $$e_{\omega }=\omega \;-\;R_{e}^{T}\omega_{d}$$

Differentiating Equations (14) and (15) and combining the dynamic equation of rotation equation of quadrotor, the dynamic equation of attitude error and angular velocity error can be obtained as:

(16) $${\dot{R}}_{e}=R_{e}{\hat{e}}_{\omega }$$

(17) $$I{\dot{e}}_{\omega }=M_{d}-\;\hat{\omega }I\omega \;+\;I{\hat{e}}_{\omega }R_{e}^{T}\omega_{d}-IR_{e}^{T}{\dot{\omega }}_{d}$$

The control objective to make rotation matrix converge to its desired value is equivalent to make $R_{e}$ converges to identity matrix E. Before the design of control law, one lemma is introduced to describe rotation matrix [25].

**Lemma** **1.** *According to the Euler’s Rotation Theorem, every rotation matrix is equivalent to a rotation about a fixed axis* $\omega \in {\mathbb{R}}^{3}$ *through an angle* θ∈[0,2π). *Specifically, the rotation matrix can be represented as:*

(18) $$R=e^{\hat{\omega }\theta }$$

The associated inverse map of Equation (18) is the logarithmic map, which is expressed as:

(19) $$\log(R)=\frac{\theta }{2\sin\theta }(R-R^{T})$$

When R converges to the identity matrix, log(R) converges to zero. From (19), we can deduce log(R) is mainly determined by the skew-symmetric matrix $R-R^{T}$. 

According to the analysis above, we define vector form of attitude error as:

(20) $$e_{R}=\varepsilon {\left(R_{e}-R_{e}^{T}\right)}^{\vee }$$

where ε is positive constant. 

Then, we define sliding mode surface based on $e_{R}$ and $e_{\omega }$ as follows:

(21) $$s=\beta e_{R}+e_{\omega }$$

Next, we design the control law rendering $\dot{s}$ satisfies:

(22) $$\dot{s}=-\lambda s-\gamma sgn\;(s)$$

where β, λ, γ are positive diagonal matrixes. 

Differentiating Equation (20) and the first time derivative of $e_{R}$ yields [11]:

(23) $${\dot{e}}_{R}=\varepsilon (tr(R_{e})I-R_{e}^{T})e_{\omega }$$

Differentiating s with respect to time and calling Equations (17) and (23):

(24) $$\begin{array}{ll} \dot{s} & =\beta {\dot{e}}_{R}+{\dot{e}}_{\omega }\\ & =\beta \varepsilon (tr(R_{e})I-R_{e}^{T})e_{\omega }+I^{-1}(M-\hat{\omega }I\omega +I{\hat{e}}_{\omega }R_{e}^{T}\omega_{d}-IR_{e}^{T}{\dot{\omega }}_{d}) \end{array}$$

Equating Equation (22) with Equation (24), the moment vector can be obtained as:

(25) $$M=I(-\beta \varepsilon (tr(R_{e})I-R_{e}^{T})e_{\omega }+\hat{\omega }I\omega -I{\hat{e}}_{\omega }R_{e}^{T}\omega_{d}+IR_{e}^{T}{\dot{\omega }}_{d}+I(-\lambda s-\gamma sgn(s))$$

Similar to stability proof of position loop, the stability of system can be easily proved. By Equation (21), when s=0, we have:

(26) $$\beta e_{R}+e_{\omega }=0$$

By Equations (23) and (26), we have the following equation:

(27) $${\dot{e}}_{\omega }=-\beta \varepsilon (tr(R_{e}^{T})I-R_{e}^{T})e_{\omega }$$

Then $e_{\omega }$ will exponentially converge to zero. Since $e_{\omega }$ converge to zero, it follows that $e_{R}$ will converge to zero according to Equation (26). 

The inner-outer loop structure makes the controllers easy to design and to tune. However, closed-loop system stability can’t be guaranteed due to coupling between the inner and outer loop. The closed-loop stability analysis of a system in cascade has been studied [26]. The global stability analysis theorems of a quadrotor based on the modified Rodrigues parameters (MRPs) error model have been proposed in [27]. Theorem 1 is adopted to demonstrate the global stability of proposed control scheme.

**Theorem** **1.** Consider the quadrotor error model in [27], if Assumption A1–A3 are satisfied, then the closed-loop system is globally stable.

A1. The control inputs $R_{d}$ and T stabilize the outer loop exponentially.

A2. The control input M stabilizes the inner loop exponentially.

A3. The control input T satisfies the condition $T\leq \frac{m}{4}(\alpha_{1}+\alpha_{2}\left\|z_{1}\right\|)$, where $\alpha_{1}$ and $\alpha_{2}$ are positive constants, $z_{1}={\left(e_{p}^{T},e_{v}^{T}\right)}^{T}$ is general error of the outer loop.

Combining the translational dynamics and Equations (16) and (17), the error model based on the rotation matrix of the quadrotor can be expressed as:

(28) $$\left\{ \begin{array}{l} {\dot{e}}_{p}=e_{v}\\ {\dot{e}}_{v}={\ddot{p}}_{d}+gz_{w}+\frac{1}{m}K\dot{p}-\frac{1}{m}RTz_{w}\\ {\dot{R}}_{e}=R_{e}{\hat{e}}_{\omega }\\ {\dot{e}}_{\omega }=I^{-1}[-\omega \times I\omega +M]-(R^{T}R_{d}{\dot{\omega }}_{d}-{\hat{e}}_{\omega }R^{T}R_{d}\omega_{d}) \end{array}\right.$$

The MRPs is another attitude representation. The attitude error $R_{e}$ is the corresponding attitude matrix of error of MRPs. The error of MRPs converges to zero only when $R_{e}$ converges to the identity matrix. The dynamics characteristics of the outer and inner loop are basically the same. The coupling term between the inner and outer loop is $\frac{1}{m}(E-RR_{d}^{T})R_{d}Tz_{w}$, which is the same as that in [27]. Therefore, Theorem 1 can be used to prove the closed-loop stability of the proposed control scheme. 

From the stability analysis of inner and outer loop, we know the proposed controllers can stabilize two loops exponentially. Based on Equation (10), the control input T can be rewritten as:

(29) $$T=\left\|m(c{\dot{e}}_{p}+ks+\eta sgn(s)+{\ddot{p}}_{d})+mgz_{w}+K\dot{p}\right\|$$

Using the properties of vector norm, T satisfies:

(30) $$\begin{array}{ll} T & \leq m(g+\left\|\eta sgn(s)+{\ddot{p}}_{d}+K\dot{p}/m\right\|)+(m\kappa )\left(\left\|e_{p}\right\|+\left\|e_{v}\right\|\right)\\ & =mg+m(\left\|\eta sgn(s)\right\|+\left\|{\ddot{p}}_{d}\right\|+\left\|K\dot{p}/m\right\|)+(m\kappa )\left(\left\|e_{p}\right\|+\left\|e_{v}\right\|\right) \end{array}$$

where κ=max(c+k,kc)η is a positive diagonal matrix from the above definition. Thus, η can be expressed by $diag(\eta_{1},\eta_{2},\eta_{3})$ where $\eta_{1}$, $\eta_{2}$ and $\eta_{3}$ are positive numbers. Based on the definition of sign function, $\left\|\eta sgn(s)\right\|=\sqrt{\eta_{1}^{2}+\eta_{2}^{2}+\eta_{3}^{2}}$. It is obvious that $\sqrt{\eta_{1}^{2}+\eta_{2}^{2}+\eta_{3}^{2}}$ is bounded with $\sqrt{\eta_{1}^{2}+\eta_{2}^{2}+\eta_{3}^{2}}\leq \mu_{1}$. The desired acceleration is bounded with $\left\|{\ddot{p}}_{d}\right\|\leq \mu_{2}$. The actual velocity $\dot{p}$ is also bounded, hence $\left\|K\dot{p}/m\right\|\leq \mu_{3}$. Where $\mu_{1}$, $\mu_{2}$ and $\mu_{3}$ are positive constants. We render μ satisfies:

(31) $$\mu =\mu_{1}+\mu_{2}+\mu_{3}$$

Subsequently, we have:

(32) $$T\leq m(g+\mu )+(m\kappa )\left(\left\|e_{p}\right\|+\left\|e_{v}\right\|\right)$$

Besides, the square of T satisfies:

(33) $$T^{2}\leq 2m^{2}{\left(g+\mu \right)}^{2}+4m^{2}\kappa^{2}({\left\|e_{p}\right\|}^{2}+{\left\|e_{v}\right\|}^{2})$$

Therefore, we have the following equation:

(34) $$\begin{array}{ll} T & \leq \sqrt{2}m(g+\mu )+2m\kappa \left\|z_{1}\right\|\\ & =\frac{m}{4}(4\sqrt{2}(g+\mu )+8m\kappa \left\|z_{1}\right\|) \end{array}$$

The control input T satisfies the assumption A3 from Equation (34). The designed controllers can stabilize the inner loop and outer loop exponentially after a finite time based on the above stability proof of the dual loop, respectively. Above all, the designed controllers satisfy all assumptions in a finite time in Theorem 1. Thus, the closed-loop stability of the system is proved. 

## 4. Simulations

In this section, the simulations are performed on Matlab/Simulink in order to test the validity of the proposed controller. The quadrotor model parameters are listed in Table 1. 

### 4.1. Parameter Selection

Heuristic method is used to select control parameters of SMC. Considering similar control laws are adopted in the inner and outer loop, we take the inner loop as an example. The controller is required to track the following rotation matrix:

(35) $$R_{d}=\left(\begin{array}{ccc} \cos(\pi t) & 0 & -\sin(\pi t)\\ \sin(\pi t) & 0 & \cos(\pi t)\\ 0 & 1 & 0 \end{array}\right)$$

**Figure 3:**
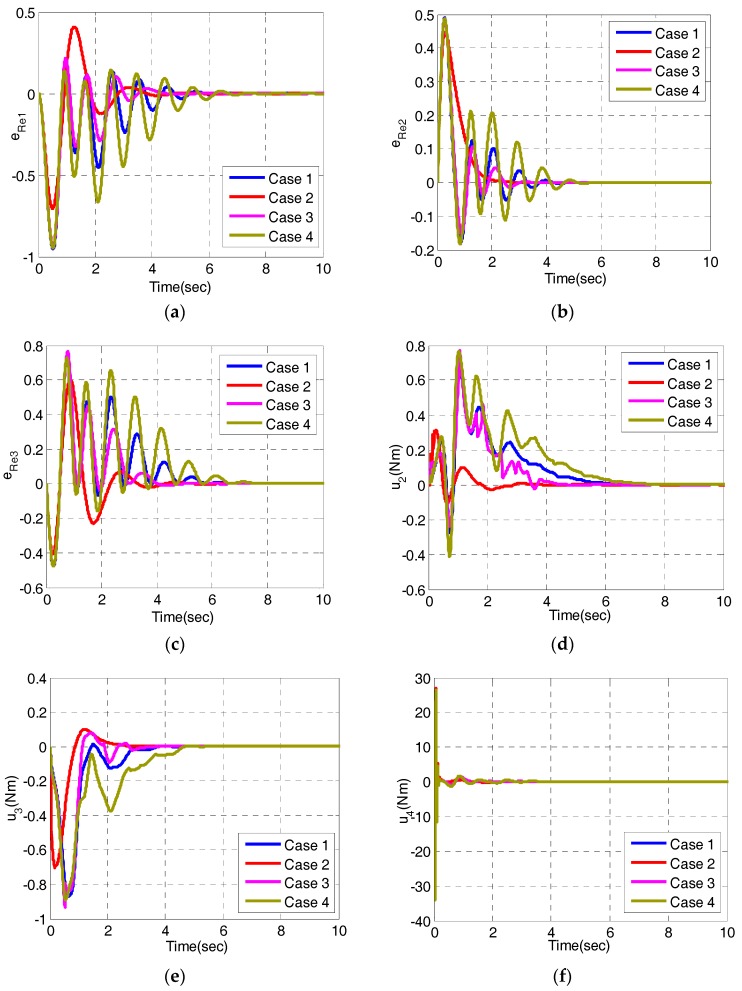
Tracking rotation matrix simulation (**a**) $e_{R_{e1}}$; (**b**) $e_{R_{e2}}$; (**c**) $e_{R_{e3}}$; (**d**) The control input $u_{2}$; (**e**) The control input $u_{3}$; (**f**) The control input $u_{4}$.

Firstly, the parameters are required to be positive to guarantee the stability of the system according to design guidelines. The effects of each parameter on the system are investigated with four sets of parameters presented in Table 2. The simulation results are shown in Figure 3. Figure 3a–c depict the vector form of attitude error and Figure 3d–f depict the control inputs. 

The quality of system is further evaluated by integral absolute error (IAE), integral squared error (ISE) and measured integral squared input (ISCI) performance index, respectively. The performance indices are summarized in Table 3. 

It is obvious that β affects the convergence rate of the system from Equation (21). We can see from Case 1 and Case 4 that the transient response deteriorates and control inputs increase when β increases. Comparing Case 1 and Case 2, the IAE, ISE and ISCI decreases when λ increases. γ is the gain of the sign function. The increase in γ makes the system performance gets better with less control inputs from Case 1 and Case 3. In conclusion, Case 2 is the most appropriate one in terms of control performance and control inputs.

### 4.2. Trajectory Tracking Simulation

#### 4.2.1. Trajectory Tracking

**Figure 4:**
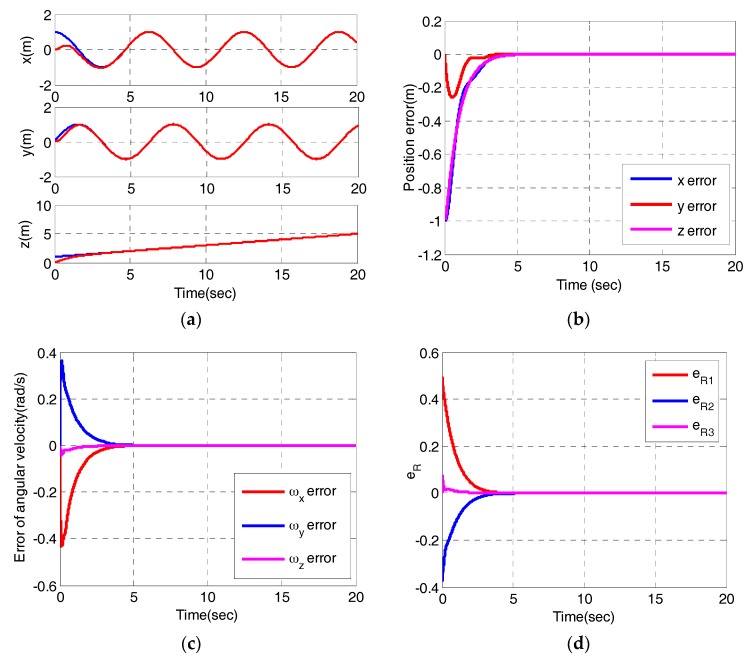

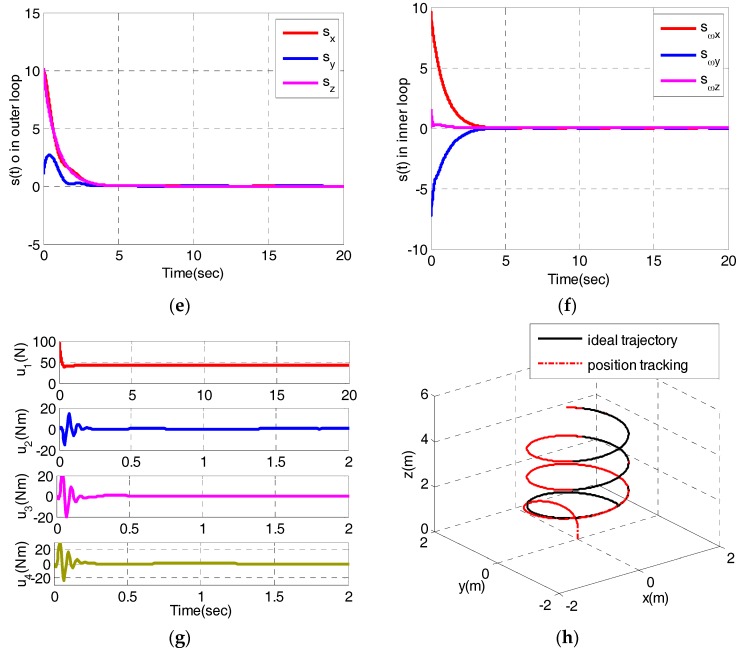
Tracking helical trajectory simulation (**a**) Position; (**b**) Error of position; (**c**) Error of angular velocity; (**d**)Vector form of attitude error; (**e**) The sliding variables in outer loop; (**f**) The sliding variables in inner loop; (**g**) The control variables; (**h**) The desired trajectory and the actual trajectory in the simulation.

In order to verify that the quadrotor can achieve trajectory tracking, three representative trajectories are simulated, respectively. The first reference trajectory is $p_{d}={\left(\cos(t),\sin(t),1+t/5\right)}^{T}$ and the desired yaw angle is πt. The initial state of the quadrotor is set to be r(0)=(0,0,0) and ψ(0)=0. The simulation results of helix tracking are shown in Figure 4. 

From Figure 4a,b, we can observe that x and z converge to their desired values without oscillation in 4 s, y converges to its desired value after 4 s later although there exists a small fluctuation. Figure 4c,d depict the errors of angular velocity $e_{\omega }$ and errors of vector form of attitude error $e_{R}$. Figure 4c demonstrates the attitude controller can track the desired angular velocities. As shown in Figure 4d, $e_{R}$ converges to zero within about 4s, which means the rotation matrix reaches the desired rotation matrix. Figure 4e,f display the behaviors of the sliding variables in the outer loop and inner loop. Obviously, all the sliding variables converge to their sliding surfaces within 4 s. Figure 4g shows the control inputs, whereby, $u_{1}$ converges to its steady state after several seconds. $u_{2}$, $u_{3}$ and $u_{4}$ converge to zero after an oscillation within 0.5 s. Figure 4h depicts the the 3-dimensional trajectory. The results demonstrate the quadrotor can realize trajectory tracking. 

**Figure 5:**
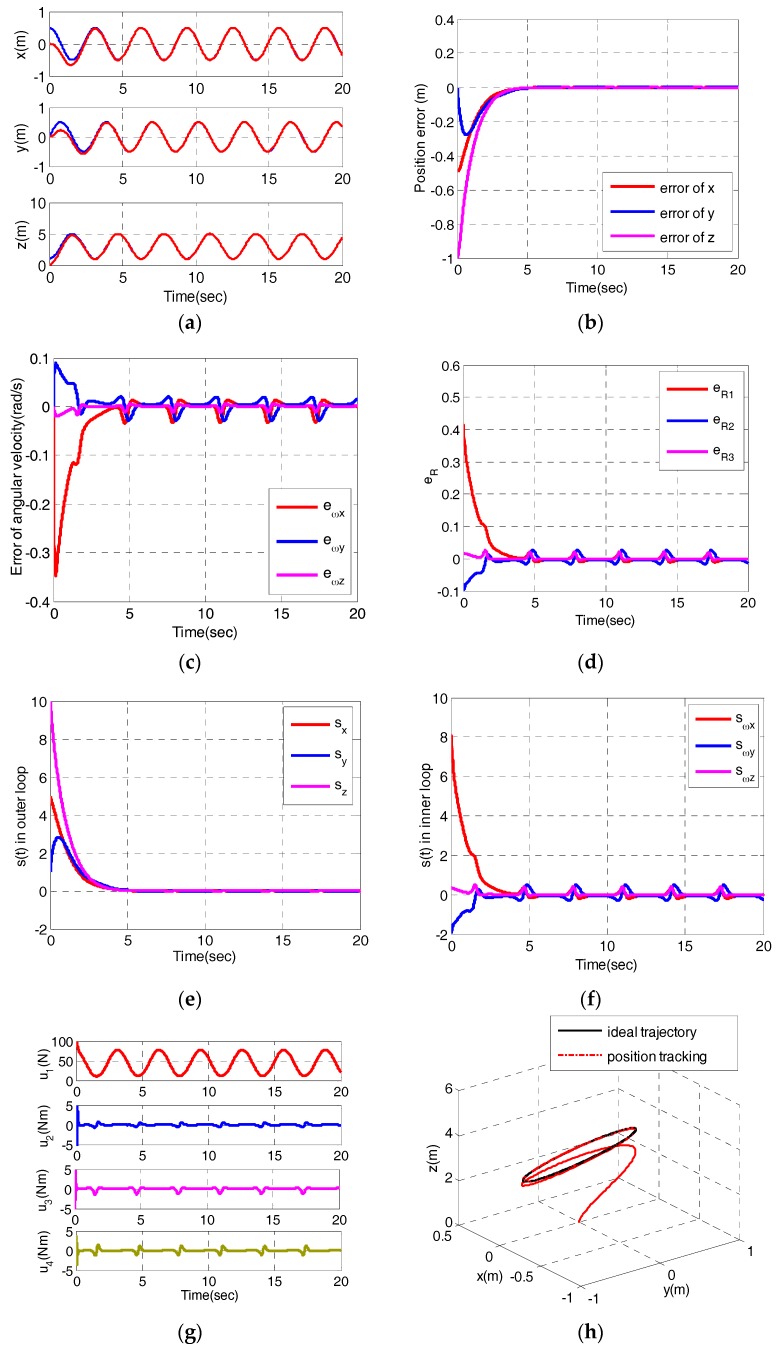
Tracking circular trajectory simulation (**a**) Position; (**b**) Error of position; (**c**) Error of angular velocity; (**d**) Vector form attitude error; (**e**) The sliding variables in outer loop; (**f**) The sliding variables in inner loop; (**g**) The control variables; (**h**) The desired trajectory and the actual trajectory in the simulation.

In order to indicate that the quadrotor can track a nontrival trajectory, an edge-on circle is chosen as another reference trajectory. Besides, the desired yaw angle is 0. The initial state of the quadrotor is set to be r(0)=(0,0,0) and ψ(0)=0. Figure 5 shows the simulation results. 

We can see from Figure 5a,b that the position can converge to its desired values soon. Figure 5c,d are the error of angular velocity $e_{\omega }$ and vector form of attitude error $e_{R}$. The great changes of angular velocity occur when the attitudes of quadrotor changes greatly. Figure 5e,f show the sliding surfaces of the outer loop and inner loop. The sliding variables of the position loop reach the sliding mode surfaces smoothly, whereas, the sliding variables of the attitude loop have a similar variation trend to that of the error of angular velocity. Figure 5g depicts the corresponding control inputs. We can observe that $u_{1}$ periodically changes as time changes, $u_{2}$, $u_{3}$ and $u_{4}$ converge to zero soon. The great changes of moment vector occur only when the attitude changes greatly. Figure 5h depicts the desired trajectory and the actual trajectory. From Figure 5h, we can see the controller can track trajectories with large Euler angles. 

A rectangle is a common trajectory and is difficult to track due to the abrupt direction changes around the four corners. The third reference trajectory is chosen as:

(36) $$p_{d}=\left\{ \begin{array}{l} x_{d}=0,y_{d}=0,z_{d}=0.5t\;0\leq t<4\\ x_{d}=0,y_{d}=0.5(t-4),z_{d}=2\;4\leq t<8\;\\ x_{d}=0.5(t-8),y_{d}=0.5,z_{d}=2\;8\leq t<12\;\\ x_{d}=2,y_{d}=0.5(t-12),z_{d}=2\;12\leq t<16\;\\ x_{d}=2-0.5(t-16),y_{d}=0,z_{d}=2\;16\leq t<20 \end{array}\right.$$

**Figure 6:**
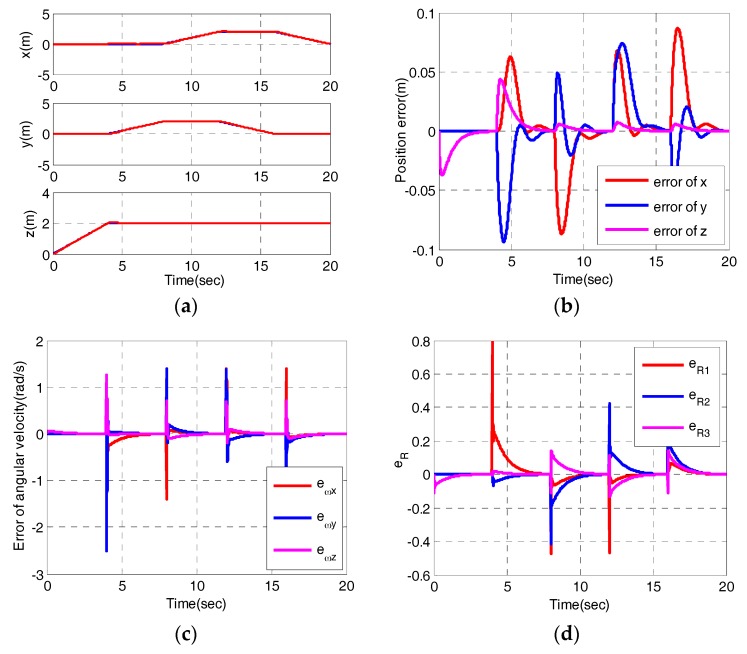

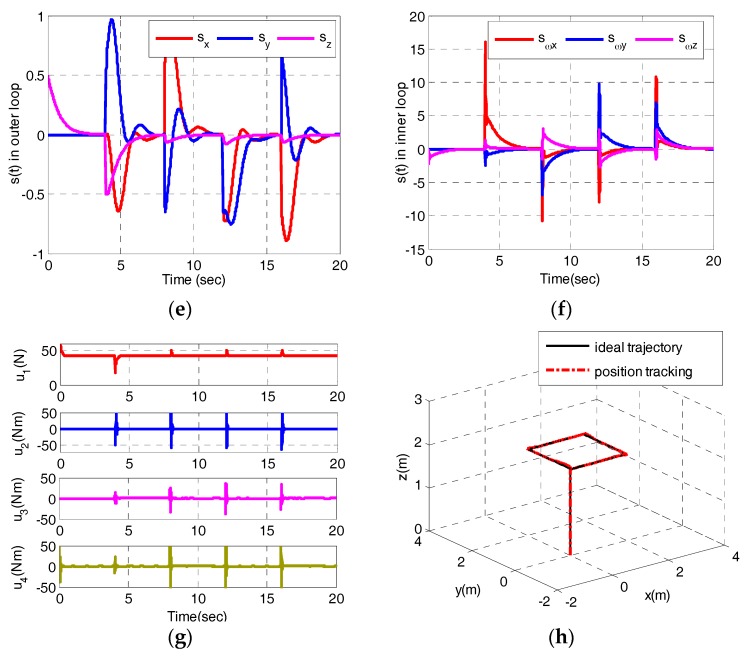
Tracking rectangular trajectory simulation (**a**) Position; (**b**) Error of position; (**c**) Error of angular velocity; (**d**)Vector form of attitude error; (**e**) The sliding variables in outer loop; (**f**) The sliding variables in inner loop; (**g**) The control variables; (**h**) The desired trajectory and the actual trajectory in the simulation.

Besides, the desired yaw is πt. The initial state of the quadrotor is set to be r(0)=(0,0,0) and ψ(0)=0. Figure 6 depicts the simulation results. 

As shown in Figure 6, the proposed control scheme can track the desired trajectory accurately after a few seconds. We can observe the position converges to its desired values soon from Figure 6a. The tracking errors reach maximum around the corner and the errors are controlled within 5 cm from Figure 6b. Figure 6c,d depict the error of angular velocity $e_{\omega }$ and vector form of attitude error $e_{R}$. As shown in Figure 6c, the errors of angular velocity monotonously reduce to zero except for jumps around the corners. The vector form of the attitude error shows similar characteristics to that of error of angular velocity. Figure 6e,f display the behaviors of the sliding variables of the outer loop and inner loop, respectively. All the sliding variables can converge to zero soon after transient transitions around the vertex of the rectangle. Figure 6g shows the control inputs. At the vertex of the rectangle, large control inputs in a short time are needed. After the vertex, $u_{1}$ converges to its steady state value, $u_{2}$, $u_{3}$ and $u_{4}$ converge to zero. The actual trajectory can track the ideal trajectory well from Figure 6h. Above all, the tracking helix demonstrates that the proposed controller can realize basic trajectory tracking. Tracking a circle and a rectangle in three-dimensional space verifies that the proposed control scheme can relieve from small angle approximation and can realize complicated flights. 

#### 4.2.2. Trajectory Tracking in Presence of Model Uncertainty and Disturbances

**Figure 7:**
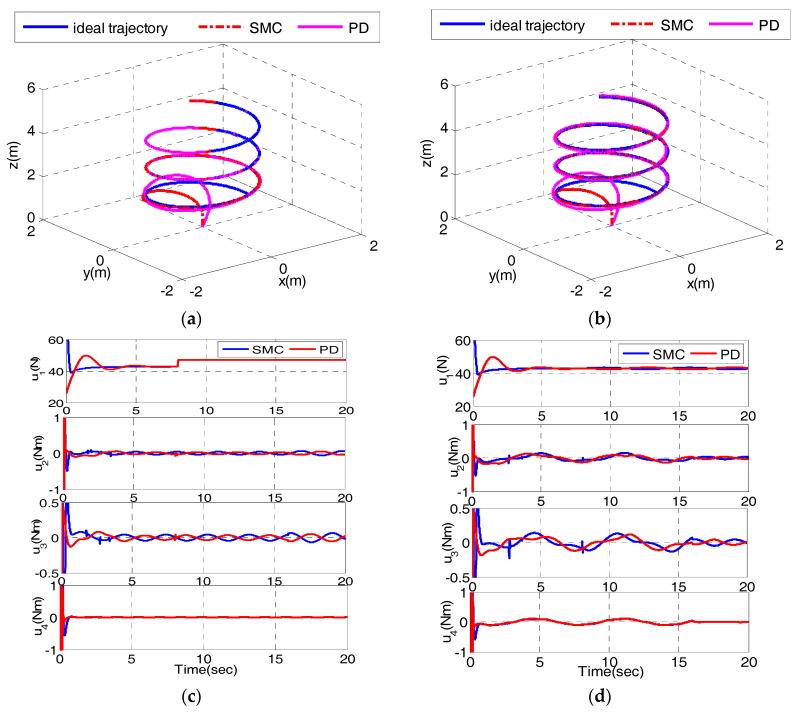
Comparisons under both controllers for trajectory tracking simulation in two cases (**a**) The trajectory in Case 1; (**b**) The trajectory in Case 2; (**c**) The control inputs in Case 1; (**d**) The control inputs in Case 2.

In order to verify the proposed control scheme is robust to parameter uncertainty and immune to bounded disturbances, two corresponding simulation experiments have been carried out [28]. As a contrast, PD and PD control combination is simulated under the same conditions [11]. In the first simulation (Case 1), we induce 0.5 m and $0.1I_{i}$ (i=1−3) to the quadrotor at time t=8s and t=16s, respectively. In the second simulation (Case 2), bounded disturbance 0.1sin(t) is added to the inner loop at time t=8s and 0.1 cos(t) is added to the outer loop at t=16s (Case 2). The simulation results are given in Figure 7. 

Figure 7a,c depict the trajectory and control inputs in Case 1. Both controllers have a good tracking performance, even in presence of model uncertainty from Figure 7a. As shown in Figure 7c, when additional mass is added to the system at time t=8s, $u_{1}$ under both controllers increases to the same value to compensate for the change of model. When the moment of inertia changes at time t=16s, $u_{2}$ and $u_{3}$ adjust to new values to cancel the effects of changes of moment of inertia. Figure 7b,d depict the trajectory and control inputs in Case 2. Figure 7b shows the tracking performance is not influenced by disturbances. The disturbances occurring at time t=8s and t=16s are both rejected by rapid adjustments of control inputs from Figure 7d. In addition, MAE, IAE and ISE are utilized to evaluate the performance of control method. Table 4 shows the quantitative values.

As shown in Table 4, MAE along the x direction occurs in the initial state and MAE with the help of both controllers is the same in Case 1. MAE along the other two directions, IAE and ISE are also less than those of PD and PD combination. It can be found that the combination of SMC and SMC has less tracking errors. While adding external disturbances to the system in Case 2, the tracking results are similar to the results in Case 1. The MAE along the x direction is the same and other performance indices are less than those of PD and PD combination. In sum, the proposed controller shows a better tracking performance in two cases, which verifies the robustness of the designed control scheme in presence of model uncertainty and disturbances. 

## 5. Experimental Results

### 5.1. Experimental Setup

**Figure 8:**
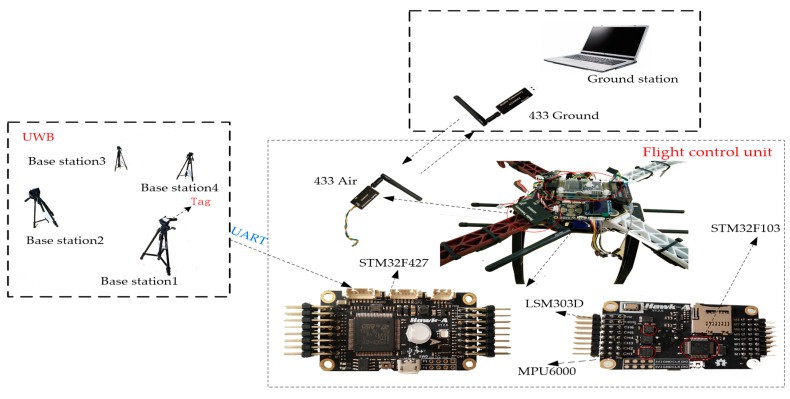
The experimental system.

The validity of the proposed method is verified using the system in Figure 8. The IMU provides angular speed, acceleration and magnetic field information for the system to estimate the pose of the quadrotor. The specifications of sensors used are listed in Table 5. The UWB system is used to provide position with an accuracy within 10 cm, which is transmitted to flight control unit via a serial port. The flight control unit consists of two embedded processors. The high level STM32F427processor (STMicroelectronics, Geneva, Switzerland) receives commands from the ground station, runs the control procedure and gives the pwm signals to motors. The low level processor STM32F103 (STMicroelectronics, Geneva, Switzerland) gives the measurement information to the STM32F427. Besides, various information including attitude angles, position and sensor information is transmitted to base station to display by wireless data transmission module 433. 

**Figure 9:**
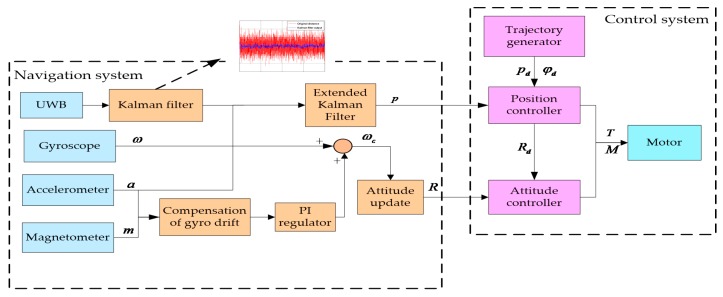
The flow diagram of the algorithm of the system.

The system is divided into two parts in Figure 9. A traditional GPS positioning device is replaced by a UWB to realize indoor positioning. Meanwhile, the position can be obtained using the acceleration and angular rate provided by an accelerometer and gyroscope through the strapdown inertial navigation algorithm. Nevertheless, IMU measurements have significant drift errors over a long time. Therefore, EKF is adopted to fuse both to get a more precise position. The rotation matrix is acquired by a complementary filter. The gyroscope is compensated by the accelerometer and magnetometer. The control system has been introduced in Section 2 and Section 3. 

**Figure 10:**
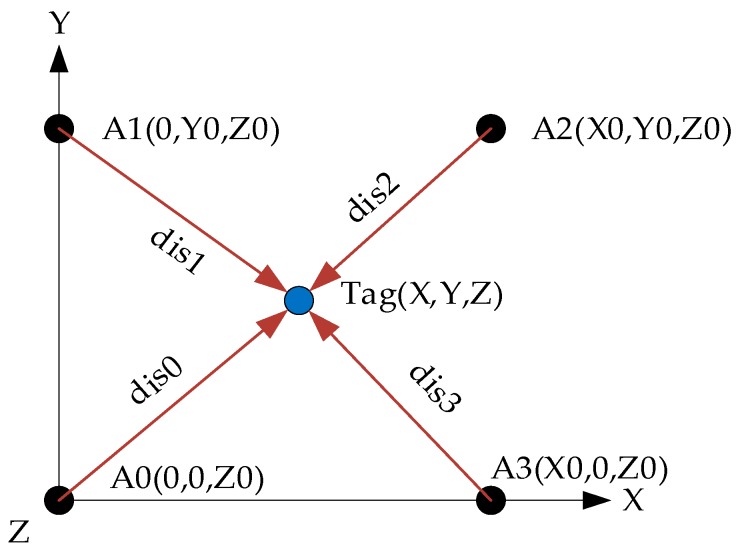
The placement of base stations and the tag.

The UWB system is produced by INF Company (Harbin, China). The placement of base stations is shown as Figure 10. 

The positions of base station (A0∼A3) are fixed and the tag is attached to the quadrotor. The tag provides the distances from the tag to each base station. Based on spatial relationship, Equation (37) can be obtained as:

(37) $$\begin{array}{l} {\left(0-X\right)}^{2}+{\left(0-Y\right)}^{2}+{\left(Z0-Z\right)}^{2}=dis0^{2}\\ {\left(0-X\right)}^{2}+{\left(Y0-Y\right)}^{2}+{\left(Z0-Z\right)}^{2}=dis1^{2}\\ {\left(X0-X\right)}^{2}+{\left(Y0-Y\right)}^{2}+{\left(Z0-Z\right)}^{2}=dis2^{2}\\ {\left(X0-X\right)}^{2}+{\left(0-Y\right)}^{2}+{\left(Z0-Z\right)}^{2}=dis3^{2} \end{array}$$

In order to obtain more accurate position, the measured distances are processed by Kalman filter to eliminate the measurement noises. The state equation and measurement equation are as follows:

(38) $$\begin{array}{l} dis_{k+1}=dis_{k}+\xi_{k}\\ dis_{k}^{m}=dis_{k}+\upsilon_{k} \end{array}$$

where $dis_{k}={\left(dis0,dis1,dis2,dis3\right)}_{k}^{T}$ is the distance vector between tag and each base station .The subscript k denotes kth sampling time. $\xi_{k}$ is process noise. $dis_{k}^{m}$ is the distance measured from UWB $\upsilon_{k}$ is measurement noise. In the experiment,$E(\xi \xi^{T})=diag(0.0001,0.0001,0.0001,0.0001)$, $E(\upsilon \upsilon^{T})=diag(0.018,0.018,0.018,0.018)$. 

The position can be determined by any three equations of Equation (34). Generally, the base station A4 is regarded as standby station. When a failure happens to one station, the station is used to calculate the position. After the Kalman filter, the position of tag can be determined as follows by solving Equation (39):

(39) $$\begin{array}{l} X=\frac{dis1^{2}-dis2^{2}+X0^{2}}{2X0}\\ Y=\frac{dis0^{2}-dis1^{2}+Y0^{2}}{2Y0}\\ Z=\left\{ \begin{array}{l} \sqrt{dis0^{2}-X^{2}-Y^{2}}+Z0\;Z>Z0\\ Z0-\sqrt{dis0^{2}-X^{2}-Y^{2}}\;Z\leq Z0 \end{array}\right. \end{array}$$

**Figure 11:**
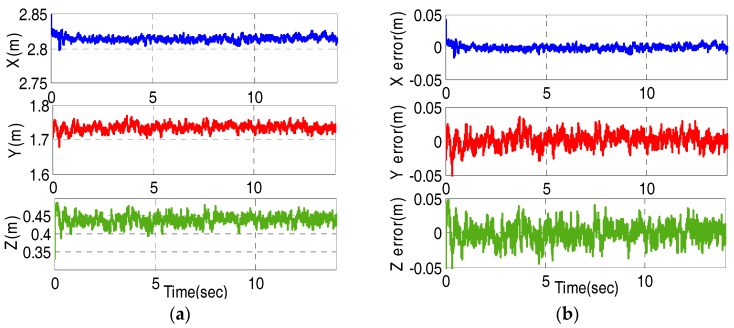
The measured position by UWB system and the corresponding errors (**a**) The measured position by UWB system; (**b**) The corresponding position errors.

In practice, we have tested the measurement accuracy of UWB system when the tag is placed in ${\left(2.81,1.73,0.43\right)}^{T}$. Figure 11 shows actual position measured by UWB system and the corresponding errors. 

We can observe that the measurement errors of UWB system are within 5 cm from Figure 11b. Generally, the inertial measurements contain a certain bias b and white Gaussian noise w. The measurement models of angular velocity and acceleration yield [29,30]:

(40) $$\begin{array}{l} \omega_{m}=\omega +b_{g}+w_{g}\\ a_{m}=R^{T}(a+g)+b_{a}+w_{a} \end{array}$$

where $\omega_{m}$ and $a_{m}$ are the measurements of gyroscope and accelerometer, respectively. ω and a are true angular velocity and acceleration in the world frame, respectively. $w_{g}$ is gyro noise with covariance $E(w_{g}w_{g}^{T})=\sigma_{w_{g}}^{2}E$ and $w_{a}$ is accelerometer noise with covariance $E(w_{a}w_{a}^{T})=\sigma_{w_{a}}^{2}E$. In addition, the gyroscope bias $b_{g}$ and accelerometer bias $b_{a}$ are modeled as random walk as:

(41) $$\begin{array}{l} {\dot{b}}_{a}=w_{b_{a}}\\ {\dot{b}}_{g}=w_{b_{g}} \end{array}$$

where $w_{b_{a}}$ and $w_{b_{g}}$ are white noise. Whereby $E(w_{b_{g}}w_{b_{g}}^{T})=\sigma_{w_{b_{g}}}^{2}E$ and $E(w_{b_{a}}w_{b_{a}}^{T})=\sigma_{w_{b_{a}}}^{2}E$. 

According to Newton’s law and Euler formula, the discrete-time kinematics equations are illustrated as follows:

(42) $$\begin{array}{l} p_{k+1}=p_{k}+v_{k}T+a_{k}T^{2}/2\\ v_{k+1}=v_{k}+a_{k}T \end{array}$$

where $a_{k}$ denotes $R_{k}(a_{mk}-b_{ak}-w_{ak})-g$ and T is the sampling period. 

For the representation of rotation matrix we use quaternions, the quaternion representation of rotation matrix is given by:

(43) $$R=\left(\begin{array}{ccc} q_{0}^{2}+q_{1}^{2}-q_{2}^{2}-q_{3}^{2} & 2(q_{1}q_{2}-q_{0}q_{3}) & 2(q_{1}q_{3}+q_{0}q_{2})\\ 2(q_{1}q_{2}+q_{0}q_{3}) & q_{0}^{2}-q_{1}^{2}+q_{2}^{2}-q_{3}^{2} & 2(q_{2}q_{3}-q_{0}q_{1})\\ 2(q_{1}q_{3}-q_{0}q_{2}) & 2(q_{2}q_{3}+q_{0}q_{1}) & q_{0}^{2}-q_{1}^{2}-q_{2}^{2}+q_{3}^{2} \end{array}\right)$$

Based on the differential equation of quaternion, the prediction of quaternion is performed as:

(44) $$q_{k+1}=(E+1/2\Omega (\omega_{mk}-b_{gk}-w_{gk})T)q_{k}$$

where:

(45) $$\Omega (\omega )=\left(\begin{array}{cccc} 0 & -\omega_{x} & -\omega_{y} & -\omega_{z}\\ \omega_{x} & 0 & \omega_{z} & -\omega_{y}\\ \omega_{y} & -\omega_{z} & 0 & \omega_{x}\\ \omega_{z} & \omega_{y} & -\omega_{x} & 0 \end{array}\right)$$

Because IMU measurements have significant drift errors over a long time, EKF is utilized to fuse measurements from IMU and measurements from UWB to get more precise position. The design of EKF refers to the design procedure in [31,32,33]. The state vector is chosen as $x_{k}={\left(p^{T},v^{T},q^{T},b_{a}^{T},b_{\omega }^{T}\right)}_{k}^{T}$. The noise vector is $w_{k}={\left(w_{a}^{T}\;w_{g}^{T}\;w_{b_{a}}^{T}\;w_{b_{g}}^{T}\right)}^{T}$. Combining Equation (39), Equation (41) and the discrete form of Equation (38), the state equation can be written as:

(46) $$\left(\begin{array}{c} \;p_{k+1}\\ v_{k+1}\\ q_{k+1}\\ b_{ak+1}\\ b_{gk+1} \end{array}\right)=\left(\begin{array}{c} p_{k}+v_{k}T+R_{k}(a_{mk}-b_{ak}-w_{ak})T^{2}/2-gT^{2}/2\\ v_{k}+R_{k}(a_{mk}-b_{ak}-w_{ak})T-gT\\ \left(E+1/2\Omega (\omega_{mk}-b_{gk}-w_{gk})T)q(k\right)\\ b_{ak}+w_{{}_{b_{a}}}T\\ b_{gk}+w_{b_{g}}T \end{array}\right)$$

The process noise covariance $Q_{k}=diag(\sigma_{w_{g}}^{2},\sigma_{w_{a}}^{2},\sigma_{w_{b_{g}}}^{2},\sigma_{w_{b_{a}}}^{2})$. The noise parameters are given by IMU manufacturer. 

The measurement of UWB is defined as $z_{k}={\left(X,Y,Z\right)}^{T}$. Then the measurement equation can be expressed as:

(47) $$z_{k}=h(x_{k})+v_{k}=Hx_{k}+v_{k}$$

where $v_{k}$ is the measurement noise. The measurement transformation matrix is $H=\left(I_{3\times 3}\;0_{3\times 13}\right)$. The observation covariance matrix $R_{k}$ can be calculated by system observations. The update of state follows the standard EKF procedure shown in Algorithm 1.

**Algorithm 1:** Extended Kalman FilterGiven the initial state $x_{0}$ and initial covariance matrix $P_{0}$, update the state estimation as follows Compute the predicted state: ${\hat{x}}_{k|k-1}=f(x_{k-1},w_{k-1})$ Compute the process model Jacobian matrix : $F_{k-1}=\frac{\partial f}{\partial x}{|}_{x_{k-1}}$
$G_{k-1}=\frac{\partial f}{\partial w}{|}_{x_{k-1}}$ Compute the predicted covariance matrix : $P_{k|k-1}=F_{k-1}P_{k-1}F_{k-1}^{T}+G_{k-1}Q_{k-1}G_{k-1}^{T}$ Compute the Kalam gain: $K_{k}=P_{k|k-1}H^{T}{\left(HP_{k|k-1}H^{T}+R_{k}\right)}^{-1}$ Update the state estimation: $x_{k}={\hat{x}}_{k|k-1}+K_{k}(z_{k}-h({\hat{x}}_{k|k-1}))$ Update the covariance matrix: $P_{k}=P_{k|k-1}-K_{k}HP_{k|k-1}$

The state of EKF updates only when the measurements from UWB system update. In practice, the inner loop run faster than the outer loop, thus the updating rate of attitude is supposed to be faster than that of the position. During the interval of state update, the attitude is obtained by the IMU and magnetometer [34]. The accelerometer and the magnetometer are utilized to calculate the error between reference direction and measured direction, which is used to compensate for the angular velocity by PI regulator. The calibrated angular velocity is further used to update quaternion according to Equation (41). The ultimate rotation matrix is obtained based on Equation (40). 

### 5.2. Experimental Results

**Figure 12:**
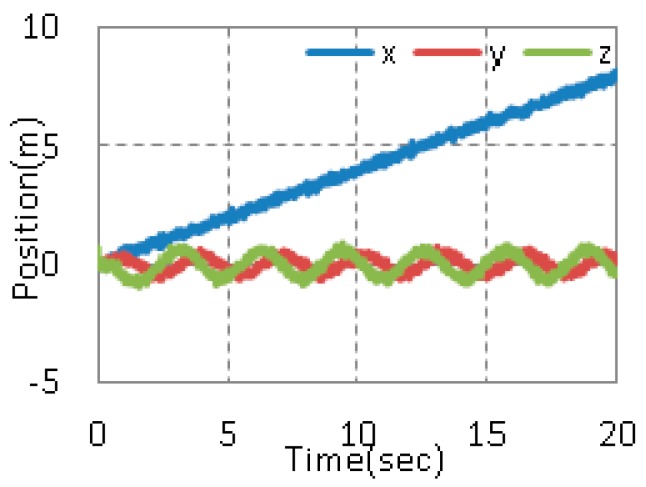
Actual position of quadrotor in the experiment.

In order to verify the validity of the tracking performance of the proposed method, we design a helical trajectory as the given trajectory. In the experiment, the outer loop runs at 100 Hz (the updating rate of UWB), while the inner loop runs at 1 kHz. Figure 12 shows the position. 

For the comparison purposes, a PD controller is employed with the same task (Case 1). The PD control parameters are tuned by a great deal of adjusting parameters. Three sets of parameters are chosen to show the effect of parameters on system. The quality of system is evaluated by maximum overshoot (MO), integral absolute error (IAE), integral squared error (ISE). The performance indices are summarized in Table 6. 

**Figure 13:**
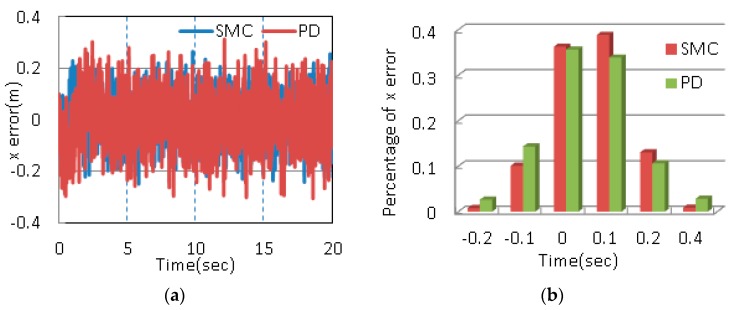

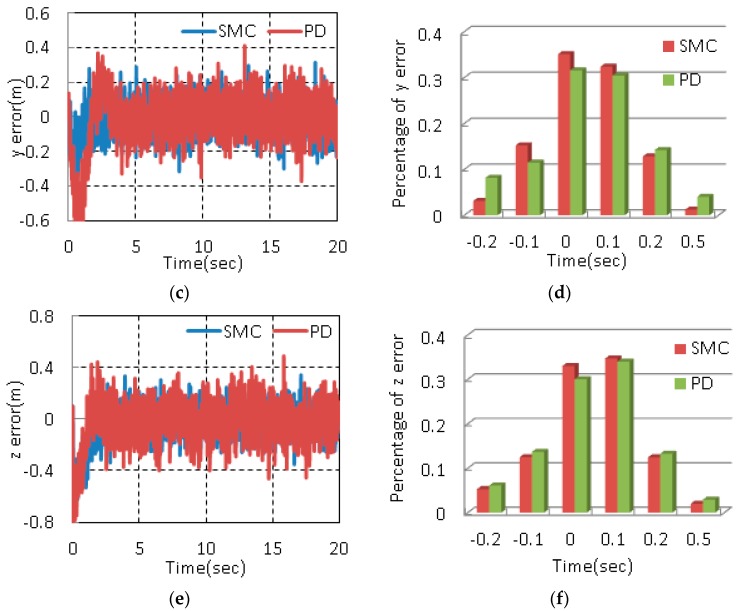
Position errors and histograms of position errors in Case 1 (**a**) x position error; (**b**) Percentage of x position error; (**c**) y position error; (**d**) Percentage of y position error; (**e**) z position error; (**f**) Percentage of z position error.

As shown in Table 6, IAE and ISE are small enough, which means the system has reached its good performance. Meanwhile, the third set of parameters has smaller MO, IAE and ISE compared with the other two sets of parameters. Thus, the third set of parameters is chosen as ultimate control parameters. Figure 13 depicts the position errors and histograms of corresponding errors. 

**Figure 14:**
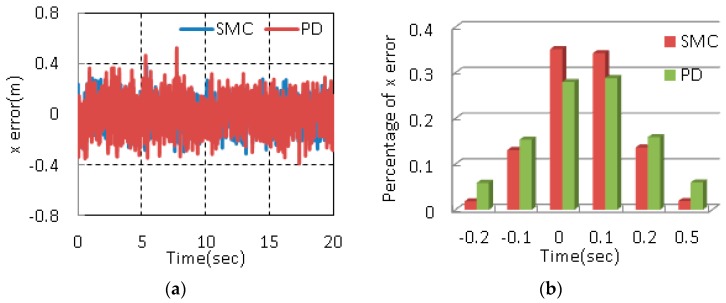

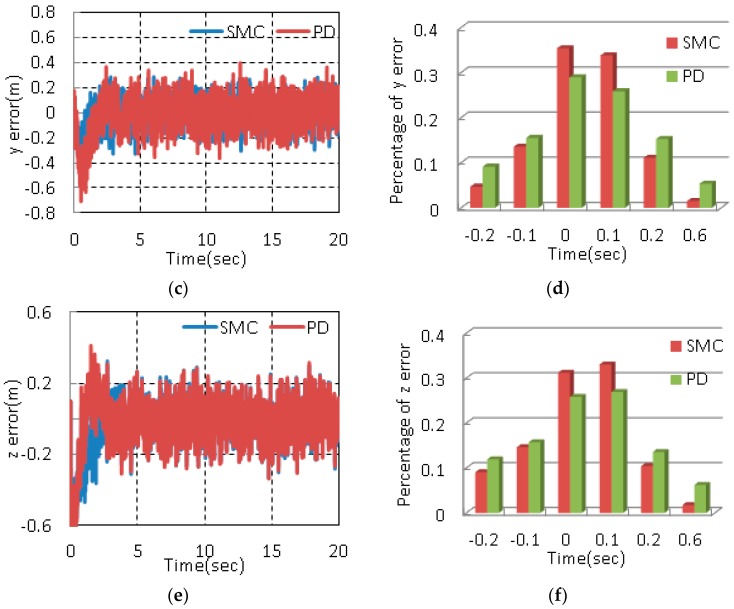
Position errors and histograms of position errors in Case 2 (**a**) x position error; (**b**) Percentage of x position error; (**c**) y position error; (**d**) Percentage of y position error; (**e**) z position error; (**f**) Percentage of z position error.

As shown in Figure 13, the position error along x is controlled within ±0.2 m, about eighty percent of the error is in the range of −0.1 m to 0.1 m. The error in y direction is in −0.4 m~0.35 m and the majority of the error is within ±0.2 m. The error for z direction is controlled within ±0.2 m, ninety percent of the error is within the range. In order to demonstrate the robustness of the proposed control method, the same experiment is carried out under disturbances (Case 2). Figure 14 shows the position errors and histograms of corresponding errors. Meanwhile, range, the percentage of error within ±0.2 m and standard deviation are adopted as quantitative performance indices to describe the control performance [35,36]. The results in the two cases are listed in Table 7. 

Fast convergence rate is one main advantages of SMC in comparison to PD control. The settling time shows the system under SMC reaches stability faster. The error range indicates that the maximum deviations of SMC are smaller than those of PD. The percentages of error demonstrate that more than 90% of the errors are within ±0.2 m under SMC. It also can be observed that only the percentage of error along x is more than 90% under PD control. Meanwhile, the percentages of error within ±0.1 m are obviously greater than those under PD control from Figure 13. The errors along y and z beyond ±0.2 m with help of PD control reach 10%, while the errors with the help of SMC are much less than 10%. The standard deviations of the designed controller are much less than those of PD controller. In other words, the proposed control scheme has better stability. Above all, the proposed control method can achieve a better tracking performance.

When the system is disturbed, the performance indices of SMC show small changes while the performance indices under PD controller obviously increase. The percentages of error within ±0.2 m with the help of SMC slightly decrease. However, the percentages of error within ±0.2 m under PD controller decrease in large amplitude. Meanwhile, the standard deviations significantly increase while the standard deviations with the help of SMC change slightly. Hence, the proposed control method shows a better performance even in presence of disturbances.

## 6. Conclusions

In this paper, a large scale dynamical model of a quadrotor is built according to the Newton-Euler equation. An inner and outer loop control method is proposed for trajectory tracking. The outer loop that governs the translational motion is designed using SMC law. A SMC inner loop controller based on a rotation matrix is designed to control the rotational motion. The dynamical model of the quadrotor with the controllers is simulated in Matlab/Simulink. The tracking helix verifies the control method has a good tracking performance. Tracking edge-on circle and rectangle trajectories further demonstrates the quadrotor can track nontrivial trajectories and realize large angle flight. The robustness of the proposed method is verified by a tracking helix in the presence of model uncertainties and external disturbances. The simulation results show that the proposed control method has better robustness compared with PD and PD combination. Based on the simulations, we conduct an experiment to further verify the feasibility of the method. The experimental results show the position is well controlled within a reasonable range. Comparative experiments with PD and PD combination further verify the effectiveness of the proposed control method. In our work, the rotation motion is realized through adjusting the rotation matrix to the desired value. The angular velocity is no longer approximated by the first time derivative of attitude angles. In this case, the quadrotor is able to track nontrivial trajectories. Meanwhile, SMC is utilized to govern the inner and outer loop. The use of SMC speeds up the convergence rate and improves the robustness of the system. Future works will focus on autonomous navigation of the quadrotor.

## Figures and Tables

**Table 1:** Quadrotor model parameters.

Name	Variable	Value	Units
Mass	M	2.0	kg
Arm length	L	0.20	m
Inertia on *x *axis	$I_{xx}$	1.25	$Ns^{2}/rad$
Inertia on *y *axis	$I_{yy}$	1.25	$Ns^{2}/rad$
Inertia on *z* axis	$I_{zz}$	2.5	$Ns^{2}/rad$
Drag coefficients	$K_{1}=K_{2}=K_{3}$	0.012	Ns/m

**Table 2:** The control parameters of the inner loop.

	$\beta_{1}$	$\beta_{2}$	$\beta_{3}$	$\lambda_{1}$	$\lambda_{2}$	$\lambda_{3}$	$\gamma_{1}$	$\gamma_{2}$	$\gamma_{3}$
**Case 1**	10	10	10	1	1	1	0.1	0.1	0.1
**Case 2**	10	10	10	5	5	5	0.1	0.1	0.1
**Case 3**	10	10	10	1	1	1	1	1	1
**Case 4**	12	12	12	1	1	1	0.1	0.1	0.1

**Table 3:** Comparison of performance of SMC with different sets of parameters.

	IAE			ISE			ISCI		
	$e_{R_{1}}$	$e_{R_{2}}$	$e_{R_{3}}$	$e_{R_{1}}$	$e_{R_{2}}$	$e_{R_{3}}$	$u_{2}$	$u_{3}$	$u_{4}$
**Case 1**	0.9682	0.3469	1.0215	0.4407	0.0845	0.3788	0.3028	0.3809	38.9353
**Case 2**	0.6981	0.3435	0.6408	0.2748	0.1024	0.2152	0.0275	0.1890	38.6488
**Case 3**	0.7641	0.2911	0.7862	0.3699	0.0791	0.3035	0.2422	0.3760	38.8503
**Case 4**	1.2993	0.4847	1.3257	0.5999	0.1073	0.5238	0.5210	0.4912	39.3407

**Table 4:** Comparisons for two control schemes in two cases.

	Index	x			y			z		
Cases		MAE	IAE	ISE	MAE	IAE	ISE	MAE	IAE	ISE
**Case 1**	PD + PD	1	1.2819	0.6831	0.5441	0.8947	0.2843	1.0052	1.1981	0.6336
	SMC + SMC	1	1.0367	0.6080	0.2608	0.3024	0.0535	1.0017	1.0085	0.5592
**Case 2**	PD + PD	1	1.6168	0.7109	0.5465	1.2783	0.3123	1.0052	1.4237	0.6357
	SMC + SMC	1	1.1433	0.6104	0.2607	0.4089	0.0541	1.0017	1.0469	0.5601

**Table 5:** Specifications of the IMU.

Sensor	Gyroscope	Accelerometer	Magnetometer
**Type**	MPU6000	MPU6000	LSM303D
**Full scale**	−1000~1000 (°/s)	−8~+8 (g)	−8~+8 (gauss)
**Sensitivity**	≤0.030 (°/s/LSB)	0.2 (mg/LSB)	≤0.320 (mGauss/LSB)

**Table 6:** Comparison of experiment results under three sets of PD parameters.

	Index	x			y			z		
Parameters		MO	IAE	ISE	MO	IAE	ISE	MO	IAE	ISE
$k_{p}$	16	0.2551	1.5721	0.1943	0.2415	2.1305	0.4862	0.1766	1.9916	0.4019
$k_{d}$	5.6									
$k_{p}$	16	0.1263	1.6070	0.2033	0.1576	2.0041	0.3983	0.1637	1.9363	0.3997
$k_{d}$	10									
$k_{p}$	18	0.1502	1.5100	0.1819	0.1074	1.9805	0.3766	0.084	1.9285	0.3883
$k_{d}$	10									

**Table 7:** Comparison of experimental results under two cases.

	Controller	SMC + SMC			PD + PD		
Index		x	y	z	x	y	z
**Case 1**	**Settling time [s]**	3.39	3.42	4.49	5.29	5.30	6.44
	**Range [m]**	(−0.25,0.26)	(−0.41,0.31)	(−0.76,0.32)	(−0.31,0.32)	(−0.72,0.41)	(−0.78,0.42)
	**The percentage of error within** ± **0.2 m**	98.3%	95.6%	92. 7%	94.3%	87.8 %	89.4 %
	**Standard deviation [m]**	0.0921	0.1006	0.1353	0.09724	0.1514	0.1396
**Case 2**	**Settling time [s]**	3.43	3.45	4.53	5.69	5.68	6.83
	**Range [m]**	(−0.31,0.35)	(−0.43,0.33)	(−0.77,0.34)	(−0.38,0.45)	(−0.73,0.46)	(−0.79,0.53)
	**The percentage of error within** ± **0.2 m**	96.2%	93.8%	90.8%	88.0%	80.4%	78.2%
	**Standard deviation [m]**	0.0935	0.1018	0.1364	0.1283	0.1698	0.1738
